# Depression, extrapyramidal symptoms, dementia and an unexpected outcome: a case report

**DOI:** 10.1186/1757-1626-3-47

**Published:** 2010-02-02

**Authors:** Magda Tsolaki, Chaido Z Messini, Marianna Siapera, Foteini Fotiadou, Dionysia Delaporta, Athanasios Karatolias

**Affiliations:** 1 3rd Department of Neurology, Aristotle University of Thessaloniki, General Hospital "G Papanikolaou", Exohi Thessaloniki, 57010, Greece; 2 Greek Association of Alzheimer's Disease and Related Disorders, 13 Petrou Sindika str, Thessaloniki, 54643, Greece

## Abstract

**Introduction:**

The diagnosis of Parkinson's disease is mainly clinical. DaT SCAN may help in difficult cases. Depression is also a clinical diagnosis and is common and persistent symptom in Parkinson's disease. Dementia is very often in Parkinson's disease, but usually not at the first stages. The treatment of each of the above symptoms is difficult and a lot of times individualized.

**Case Presentation:**

Female 64 years old patient with history of hypothyroidism, depression and anxiety disorder was examined at outpatient Memory and Dementia clinic of 3^rd ^Department of Neurology. The patient's major problems were functional and cognitive decline, severe extrapyramidal symptoms and depression. According to UKPDS Brain Bank criteria the patient had bradykinesia, muscular rigidity, postural instability and rest tremor present with unilateral onset of the symptoms affecting left side most and progressive course. The modified Hoehn and Yahr scale was 3: mild to moderate bilateral disease; some postural instability; physically independent. The symptoms remained during nine months follow up, despite the pharmaceutical treatment. Nine months later, the patient made an attempt to suicide. Firstly, she was transferred to intensive care department with 2^nd ^degree burns and respiratory problems, then she was hospitalized at the Burn Unit and afterwards at the Psychiatric clinic. One month later the patient had no depression, a clear reduction of the extrapyramidal symptoms, functional and cognitive improvement.

**Conclusion:**

An astonishing improvement occurred after the threat of life. Two years after the attempt to suicide, the depressive symptoms remain in remission and functional and cognitive status is normal. The extrapyramidal symptoms have disappeared.

## Introduction

The diagnosis of Parkinson's disease (PD) is, most of the times, clinical with sensitivity of 88% and specificity 78% [1,2]. DaT SCAN may help in difficult cases [3,4]. Depression is a common (up to 40%) and persistent symptom in Parkinson's disease [5,6]. Depression is sometimes the first symptom of PD [7]. Other authors suggest that depression is a risk factor for PD [8]. The treatment of the depression in PD is difficult because of the lack of evidence [9]. Dementia also has high prevalence in Parkinson's disease [10]. Drug induced Parkinsonism should also be considered as a possible diagnosis. It is reported to be 20% of the patients with Parkinsonism [11]. It is well known that depression is associated with cognitive decline. Pseudodementia is a term that is usually used, but does not exist as an entity to any classification system. Furthermore depression has proved to be a risk factor for dementia and elderly patients with depression should have an ongoing monitoring for their cognitive function [12].

## Case Presentation

A 64 years old Caucasian female patient, education: 6 years, with history of hypothyroidism, depression, anxiety disorder and extrapyramidal symptoms was examined at our Memory and Dementia outpatient center on 26/1/2007.

### Personal Past History

Depression with anxiety disorder started in summer of 2004. From 28/7/2004 to 22/11/2004 (4 months) the patient was taking Citalopram 20 mg per day and Alprazolam 0.5 mg three times per day. On 22/11/04 Citalopram was changed to Mirtazapine 45 mg per day and Alprazolam was changed to Prazepam 20 mg per day. On 2/11/2005 Sulpiride 50 mg three times per day was added. It is the first time that the patient takes antipsychotic treatment. Prazepam was stopped. On 4/1/2006 (after 13 months use) Mirtazapine was changed to Chlorimipramine Hydrochloride 75 mg per day and Alprazolam 2 mg per day was added, while on 17/1/2006 Risperidon 0.5 mg per day was added and Sulpiride was stopped. That treatment stayed almost the same (Risperidon was raised to 1.5 mg per day for two months, 19/7/06-21/9/06, but remained to 0.5 mg for the period 21/9/06 to 26/1/07 when the patient came to our clinic).

### Current history

#### 1^st ^visit: 26/1/07

The patient's major problems were functional and cognitive decline, severe extrapyramidal symptoms and depression. The hypothyroidism was controlled (Levothyroxin) and for depression and anxiety disorder the patient was taking Perphenazine/Amitriptyline hydrochloride 25/2 mg twice/day, Risperidon 0.5 mg/day, Prazepam 5 mg/day. According to United Kingdom Parkinson's Disease Society (UKPDS) Brain Bank criteria the patient had bradykinesia, muscular rigidity, postural instability and and rest tremor present with unilateral onset of the symptoms affecting left side most and progressive course. None of the exclusion criteria occurred. The modified Hoehn and Yahr scale was at the stage 3: mild to moderate bilateral disease; some postural instability; physically independent. The patient performed the Unified Parkinson's Disease Rating Scale (UPDRS) [4]. For the part I Mentation, Behavior and Mood the score was 7. For the part II Activities of Daily Living the score was 23. For the part III Motor Examination the score was 59. For the part IV Complications of Therapy the score was 1 (presence of early dystonia).

The neuropsychological examination showed: Mini-Mental State Examination (MMSE): 22/30, Cambridge Examination for Mental Disorders of the Elderly (CAMCOG): 49/107, Functional Rating Scale for Symptoms in Dementia (FRSSD):17, Geriatric Depression Scale (GDS):12/15, HAMILTON: 19. Escitalopram 10 mg per day was added.

#### 2^nd ^visit: 21/2/07

The neuropsychological examination of the patient showed an improvement in cognitive and functional symptoms as well as in depression: MMSE: 25, FRSSD: 8, GDS: 10, HAMILTON: 13. Risperidon gradually was reduced and stopped, Tiapride was added.

#### 3^rd ^visit: 12/4/07

The neuropsychological examination showed MMSE: 21, FRSSD: 11, Functional Cognitive Assessment Scale (FUCAS): 84, GDS: 12, HAMILTON: 26. Tiapride was stopped because there was no difference in depression and extrapyramidal signs.

#### 4^th ^visit: 20/4/07

The United Kingdom Parkinson's disease Society (UKPDS) Brain Bank criteria and the modified Hoehn and Yahr scale remained the same, as at visit 1. Carvidopa/Levodopa/entacapone (25/100/200) mg was added.

Escitalopram 10 mg was raised to three times per day for the resistant depression.

#### 5^th ^visit: 3/5/07

**Figure 1:**
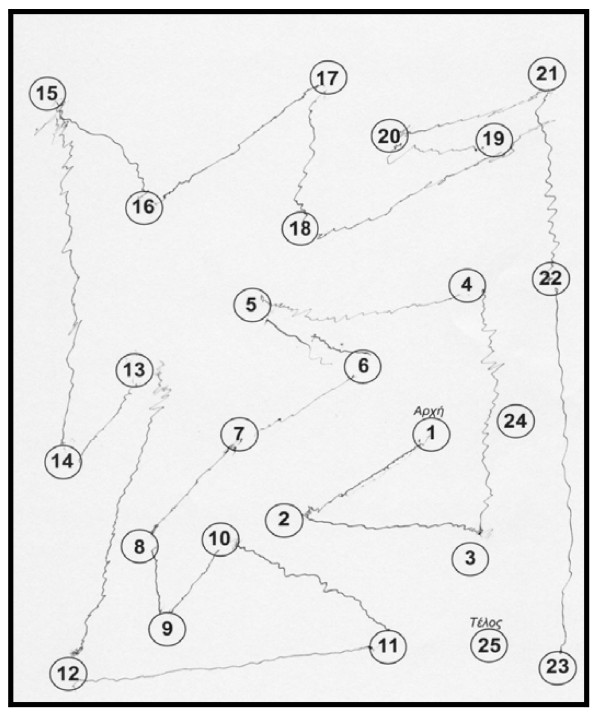
**Trail Making Test on 3^rd ^May 2007, part A, before the attempt**.

**Figure 2:**
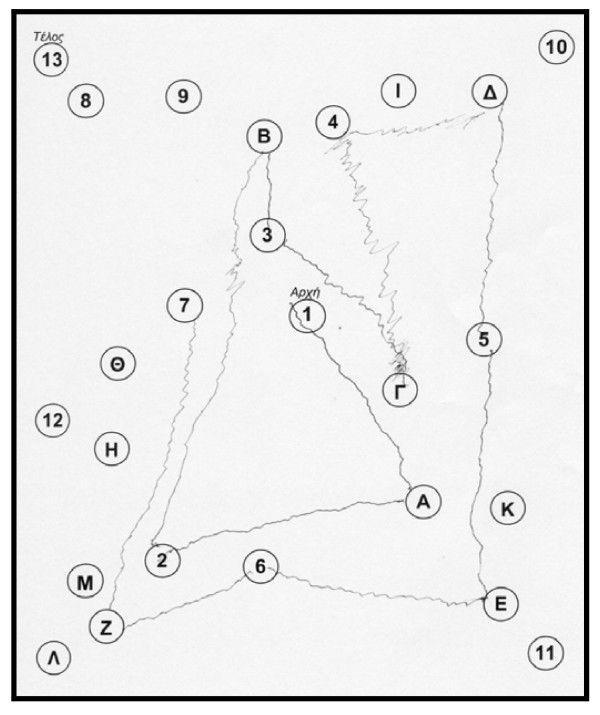
**Trail Making Test on 3^rd ^May 2007, part B, before the attempt**.

**Figure 3:**
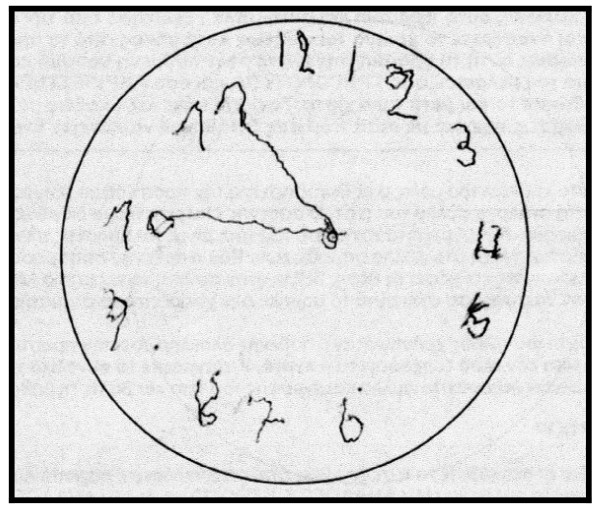
**Clock Drawing Test on 3rd May 2007, before the attempt**.

The patient performed the Unified Parkinson's Disease Rating Scale (UPDRS) and the score was the same as visit 1. The rest neuropsychological examination: Trail Making Test, part A: impaired, (figure 1) part B: impaired (figure 2). Controlled Oral Word Association Test (COWAT): 2-3-3. Boston Naming Test (BNT): 17/30. Clock Drawing Test: impaired, 3/10 (figure 3). CT was performed on 24/4/07 with no particular findings, just mild atrophy.

### An unexpected incident

On 17/09/07 the patient refused to speak and eat and had negative behavior. On 21/9/07 the patient made an attempt to suicide by starting fire. She was transferred to the local hospital to intensive care department with 2^nd ^degree burns and respiratory problems. On 9/10/07 she was transferred to the Burn Unit and on 15/10/07 to the Psychiatric Unit of our Hospital. The medication at the Psychiatric Unit was Quinapril/Hydrochlorothiazide 20 mg/day po, Carvidopa 300 mg/day po, Escitalopram 30 mg/day po, Mirtazapine 45 mg/day po, Quetiapine 200 mg/day po, Nadroparin Calcium 0.3 ml/day S.C, Iron protein succinylate 1 × 2, po, Levothyroxin 100 mg/day po.

#### 6^th ^visit: 19/10/07, one month later

The patient performed the Unified Parkinson's Disease Rating Scale.

For the part I Mentation, Behavior and Mood the score was 4

For the part II Activities of Daily Living the score was 9

For the part III Motor Examination the score was 23.5

For the part IV Complications of Therapy the score was 1(presence of early dystonia)

Hence, there was a clear reduction of the extrapyramidal symptoms.

**Figure 4:**
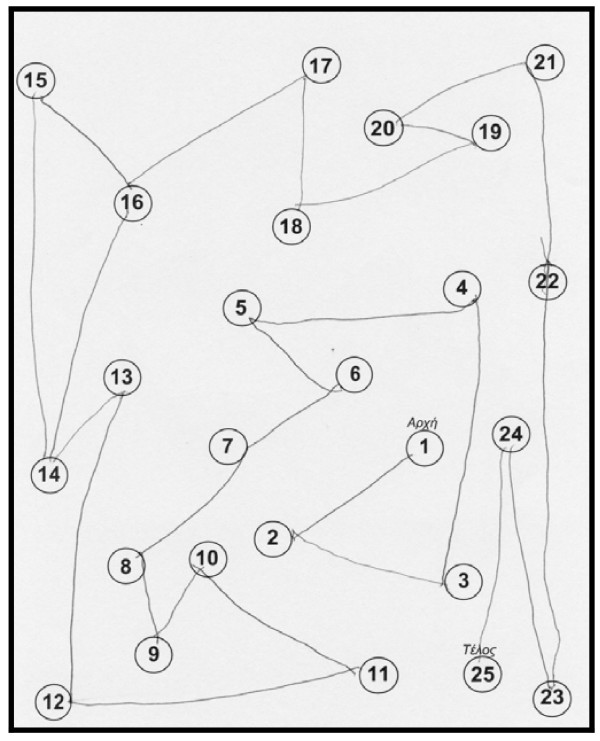
**Trail Making Test on 19^th ^October 2007, part A, after the attempt**.

**Figure 5:**
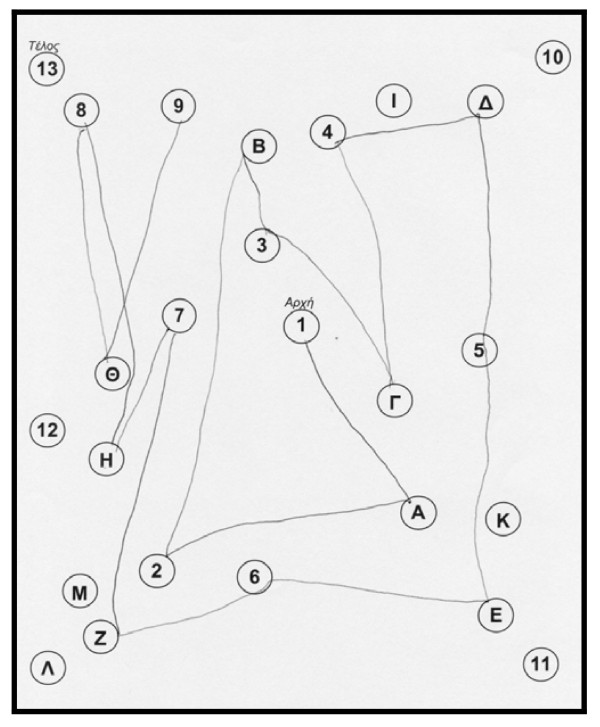
**Trail Making Test on 19^th ^October 2007, part B, after the attempt**.

**Figure 6:**
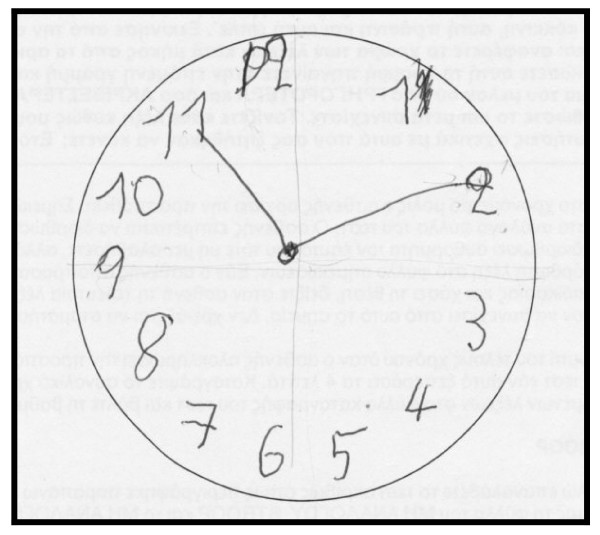
**Clock Drawing Test on 19^th ^October 2007, after the attempt**.

The neuropsychological examination showed an improvement: Trail Making Test, part A: done at 2 minutes and 18 sec, (figure 4) part B: improved (figure 5). Controlled Oral Word Association Test (COWAT): 6-5-4. Boston Naming Test (BNT): 21/30. Clock Drawing Test: 10/10 (figure 6).

The medication at that time was Mirtazapine 45 mg/day po, Escitalopram 30 mg/day po, Carvidopa/Levodopa/entacapone (25/100/200) mg S: 1*3, Levothyroxin 100 mg/day po.

The patient had no suicidal thoughts, neither depressive symptoms.

#### 7^th ^visit: 2/9/08, one year later

The patient performed the Unified Parkinson's Disease Rating Scale:

For the part I Mentation, Behavior and Mood the score was 1

For the part II Activities of Daily Living the score was 2

For the part III Motor Examination the score was 3

For the part IV Complications of Therapy the score was 0

The MMSE score was 27, the FRSSD score was 0 and the Hamilton Scale score was 4.

The UKPDS Brain Bank criteria are no longer fulfilled. The modified Hoehn and Yahr scale was at the stage 0, meaning no signs of disease.

The patient had still no suicidal thoughts, neither depressive symptoms.

#### 8^th ^visit: 12/11/08

The patient performed the Unified Parkinson's Disease Rating Scale:

For the part I Mentation, Behavior and Mood the score was 1

For the part II Activities of Daily Living the score was 0

For the part III Motor Examination the score was 5

For the part IV Complications of Therapy the score was 0

The MMSE score was 27, the FRSSD score was 0, the NPI score was 0 and the

GDS score was 0.

On **2/1/2009 **the MMSE score was 30.

On **16/1/2008**, **I-123-DaTSCAN **[3,4,13] was performed with the following findings (Table 1).

**Table 1:** I-123-DaTSCAN on 16/1/2008

Region	Right after 3.5 hours	Left after 3.5 hours	Normal ranges after 3.0 - 4.5 hours
Corpus Striatum	2.15	2.28	2.200 ± 0.30

Nucleus Caudatus - NC	2.93	3.03	2.503 ± 0.30

**Lenticular Nucleus - LN**	**0.98**	**1.03**	**2.200 ± 0.50**

Ratio LN/NC	0.33	0.34	0.88 ± 0.8

The conclusion of the I-123-DaTSCAN was that these findings (rates out of the normal range for the Lenticular Nucleus) could not exclude the start of Parkinson's disease or the start of a mild extrapyramidal disorder.

On **28/11/2008**, another **I-123-DaTSCAN **was performed with the following findings, which exclude Parkinson's disease and other Parkinsonian Syndromes (Table 2).

**Table 2:** I-123-DaTSCAN on 28/11/2008

Region	Right after 3.5 hours	Left after 3.5 hours	Normal ranges after 3.0 - 4.5 hours
Corpus Striatum	3.16	3.62	2.200 ± 0.30

Nucleus Caudatus - NC	3.86	4.32	2.503 ± 0.30

Lenticular Nucleus - LN	2.70	2.97	2.200 ± 0.50

Ratio LN/NC	0.70	0.69	0.88 ± 0.8

The **brain MRI **which was performed on 29/1/2009 was almost normal according to her age (mild periventricular leukoencephalopathy).

## Conclusion

An astonishing improvement occurred after the threat of life. The improvement is in functional cognitive and depressive symptoms. The parkinsonian symptoms also disappeared. The depressive symptoms remain in remission after a year and a half of follow up. One explanation is that the patient had Drug - Induced Parkinsonism-DIP- (Risperidon and Sulpiride), Drug induced dementia (Chlorimipramine Hydrochloride, Perphenazine/Amitriptyline hydrochloride-anticholinergic effect). On the other hand, DIP is dose-related [11] and Risperidon was given in a very low dose. Risperidon is also atypical antipsychotic, like Sulpiride which was also used previously for treating the patient. Another problem for the diagnosis of DIP is the findings of I-123-DaTSCAN on 16/1/2008 which support the diagnosis of Parkinson's disease. But this diagnosis was not clinically confirmed at the last visits, neither the 2^nd ^I-123-DaTSCAN on 28/11/08 supported it. As far as the depression's remission, it is referred at the literature that depressed patients may experience significant relief of their depressive symptoms after the suicide attempt, but there is a high probability to undergo a relapse within a short period (3 months) [14]. In this case there is no relapse so far. (Two years after the suicide attempt). A close longitudinal follow up of the patient might give us more answers.

## Consent

Written informed consent was obtained from the patient for publication of this case report and accompanying images. A copy of the written consent is available for review by the journal's Editor-in-Chief.

## Competing interests

The authors declare that they have no competing interests.

## Authors' contributions

MT, CZM, MS, FF, DD and AK participated in the evaluation and care of the patient and her caregiver. MT coordinated and directed the work. MT and MS involved in writing the manuscript. CZM, FF and DD assisted in writing the manuscript. All authors read and approved the final manuscript.

## References

[B1] Schrag A Ben-Shlomo Y Quinn N How valid is the clinical diagnosis of Parkinson's disease in the community? J Neurol Neurosurg Psychiatry 2002 73 529 534 10.1136/jnnp.73.5.529 12397145 PMC1738115

[B2] Jankovic J Parkinson's disease: clinical features and diagnosis J Neurol Neurosurg Psychiatry 2008 79 368 376 10.1136/jnnp.2007.131045 18344392

[B3] Csoti I Fornadi F Klett R Puille M Bauer R Experiences with DaTSCAN™ SPECT in the clinical practice in our Parkinson-Center Clinical Neurophysiology 2007 118 4 e22 e23 10.1016/j.clinph.2006.11.057

[B4] Manoharan P Jamieson S Bury R Initial clinical experience with [123I]ioflupane scintigraphy in movement disorders Clinical Radiology 62 5 463 471 10.1016/j.crad.2006.10.013 17398272

[B5] Prado RC Barbosa ER Depression in Parkinson's disease: study of 60 cases Arq Neuropsiquiatr 2005 63 3B 766 71 16258653 10.1590/s0004-282x2005000500009

[B6] Frisina P G Borod J C Foldi N S Tenenbaum H R Depression in Parkinson's disease: Health risks, etiology, and treatment options Neuropsychiatr Dis Treat 2008 4 1 81 91 18728814 10.2147/ndt.s1453 PMC2515908

[B7] American Academy of Neurology (April 28) Depression May Be Early Sign Of Parkinson's Disease American Academy of Neurology's 59th Annual Meeting, Boston, April 28-May 5, 2007. News release

[B8] Schuurman Increased risk of Parkinson's disease after depression: A retrospective cohort study Neurology 2002 58 1501 1504 12034786 10.1212/wnl.58.10.1501

[B9] Ghazi-Noori S Chung TH Deane KHO Rickards H Clarke CE Therapies for depression in Parkinson's disease Cochrane Database of Systematic Reviews 2003 2 CD003465 DOI: 10.1002/14651858.CD003465 10.1002/14651858.CD003465 12917968

[B10] Friedman A Barcikowska M Dementia in Parkinson's Disease Dementia 1994 5 12 16 8156081 10.1159/000106688

[B11] Hirose G Drug induced parkinsonism: a review J Neurol 2006 253 Suppl 3 III/22 III/24

[B12] Sáez-Fonseca JA Lee L Walker Z Long-term outcome of depressive pseudodementia in the elderly J Affect Disord 2007 101 1-3 123 9 10.1016/j.jad.2006.11.004 17184844

[B13] Castrejón A S Vincente García AM Cortés Romera M Vaamonde Cano J Rodado Marina S Poblete García VM Ruiz Solís S del Prado Talavera Rubio M 123-I ioflupane (Datscan^®^) presynaptic nigrostriatal imaging in patients with movement disorders Braz arch biol technol 2005 48 Special 115 125

[B14] Sakamoto K Fukunaga T The impact of attempted suicide on the symptoms and course of mood disorders J Clin Psychiatry 2003 64 10 1210 6 14658970 10.4088/jcp.v64n1011

